# Non‐Interventional Resolution of External Cervical Root Resorption: A Case Report

**DOI:** 10.1155/crid/4601457

**Published:** 2026-04-17

**Authors:** Farnam Pourreza-Jorshari, Georgios Patouris, Faraz Pourreza-Jorshari, Lee Feinberg, Shalini Kanagasingam

**Affiliations:** ^1^ Postgraduate Endodontic Unit, King’s College London Dental Institute, London, UK, kcl.ac.uk; ^2^ Postgraduate Endodontic Unit, University of Chester, Chester, UK, chester.ac.uk; ^3^ Department of Maxillofacial Radiology, King’s College London Dental Hospital, London, UK, kcl.ac.uk; ^4^ School of Medicine and Dentistry, University of Central Lancashire, Preston, UK, uclan.ac.uk

**Keywords:** diagnosis, external cervical resorption, resolution, reversal, treatment

## Abstract

External cervical resorption (ECR) is an uncommon, often asymptomatic condition that originates below the epithelial attachment. Orthodontic treatment is recognised as a common predisposing factor. This report describes a 24‐year‐old female with a mandibular second molar (Tooth 37) previously diagnosed with Heithersay Class 3 ECR. The lesion was initially deemed untreatable and managed conservatively with long‐term monitoring. After 8 years, radiographic imaging demonstrated apparent hard‐tissue deposition and lesion regression. Clinical and radiographic assessments confirmed normal pulp vitality, absence of periapical pathology, and no symptoms. Radiographic evaluation also identified an additional ECR lesion affecting the maxillary first molar (Tooth 26), highlighting the diagnostic value of thorough imaging in ECR cases. This case suggests that even advanced ECR lesions may stabilise in the absence of ongoing resorptive stimuli, supporting a cautious and individualised approach to ECR management.

## 1. Introduction

Dental resorption is characterised by the progressive loss of mineralised tooth structure mediated by clastic cell activity. External cervical resorption (ECR), a specific form of external root resorption, typically initiates just apical to the epithelial attachment in the cervical region of the tooth [1]. In certain situations, such as gingival recession or loss of periodontal support, the lesion’s entry point may be located more apically [2]. Early descriptions and classifications of ECR were provided by Heithersay, whose work remains foundational to the current understanding of the condition [3–5]. Epidemiological data suggest that ECR is relatively uncommon, with reported prevalence ranging from approximately 1.35% to 2.3% in retrospective imaging‐based studies [6, 7]. The condition most frequently involves the maxillary central incisors, followed by the maxillary canines and lateral incisors, with posterior teeth such as the mandibular and maxillary first molars affected less commonly [8].

The aetiology of ECR is multifactorial and not yet fully understood. Several predisposing factors have been proposed, including previous orthodontic treatment, dental trauma, parafunctional habits and even environmental exposures such as domestic cat ownership [9]. While many patients present with a single identifiable risk factor, a substantial proportion exhibit multiple contributing factors and a notable subset presents without any identifiable cause, highlighting the complexity of the disease process and underscoring the need for further research into the aetiopathology of this condition [9]. Among the recognised associations, orthodontic treatment has been consistently reported as one of the most common. Previous studies have identified orthodontic history in approximately 25.6%–45.7% of affected individuals, supporting a potential link between orthodontic forces and the initiation of resorptive activity [4, 10].

Management of ECR is largely dependent on the lesion’s extent, location, and accessibility. As outlined by Patel et al. [11], superficial supra‐crestal lesions with limited circumferential spread may be amenable to surgical debridement and restoration. In contrast, more extensive lesions may require additional interventions such as vital pulp therapy or root canal treatment. In cases where surgical access is restricted, internal repair in conjunction with endodontic treatment may be considered. Advanced lesions that are not amenable to conventional management strategies may necessitate more complex approaches, including intentional replantation. However, long‐term outcomes remain variable. A retrospective cohort study with up to 10 years of follow‐up reported failure rates approaching 50%, particularly in more advanced lesions such as Heithersay Classes 3 and 4 [12]. In such situations, especially when the tooth is asymptomatic and functional, periodic monitoring may be appropriate, with extraction reserved for symptomatic or non‐restorable cases.

This report describes a case of a Heithersay Class 3 ECR lesion, most likely associated with a previous history of orthodontic treatment, which was managed conservatively via monitoring due to an initially unfavourable prognosis. Unexpectedly, long‐term follow‐up demonstrated radiographic evidence suggestive of lesion regression and hard‐tissue deposition. This finding raises important questions regarding the biological behaviour of ECR and the potential for spontaneous stabilisation in the absence of ongoing stimuli, with implications for clinical decision‐making in selected cases.

## 2. Case Report

A 24‐year‐old female presented for endodontic evaluation of their mandibular left second molar (Tooth 37).

### 2.1. Reason for Referral

During a routine dental examination, a recently appointed general dentist reviewed the case of a long‐standing patient at their practice. Historical bitewing radiographs demonstrated ECR affecting the asymptomatic 37, previously diagnosed by the patient’s former dentist (Figure 1a,b). At that time, the lesion was deemed untreatable, thus, the patient and their parent consented to taking a conservative approach, involving monitoring the tooth until the onset of symptoms, at which point extraction was anticipated. As part of the current assessment, the general dentist obtained a periapical radiograph of Tooth 37 (Figure 1c). Surprisingly, the radiographic findings suggested apparent resolution of the ECR lesion. Considering these unexpected changes, the general dentist referred the patient to an endodontist for re‐evaluation of the prognosis and to determine the necessity for further intervention.

Figure 1Serial radiographic evaluation of the right maxillary and mandibular posterior region over a 7‐year period. (a) The initial bitewing radiograph taken at the patient’s general dental practice post‐orthodontic therapy, taken on 18/10/2017, shows a well‐demarcated radiolucency within the pulp chamber of Tooth 37, extending into the coronal third of both the mesial and distal roots, consistent with an external cervical resorption (ECR) lesion. The lesion was classified as Heithersay Class 3 [13]. (b) At 6 months follow‐up (11/04/2018), a review bitewing radiograph demonstrates persistence of the radiolucency, although it appears reduced in both size and radiographic density. The lesion exhibits a more diffuse and cloudy appearance, with a surrounding halo of radiopacity along its periphery, suggestive of a reparative response. (c) At the 7‐year follow‐up (12/09/2024), a periapical radiograph reveals complete resolution of the ECR lesion, with normalisation of pulp chamber dimensions and no evidence of periapical pathology. Alveolar bone levels remained stable throughout the entire 7‐year follow‐up.

**Figure:**
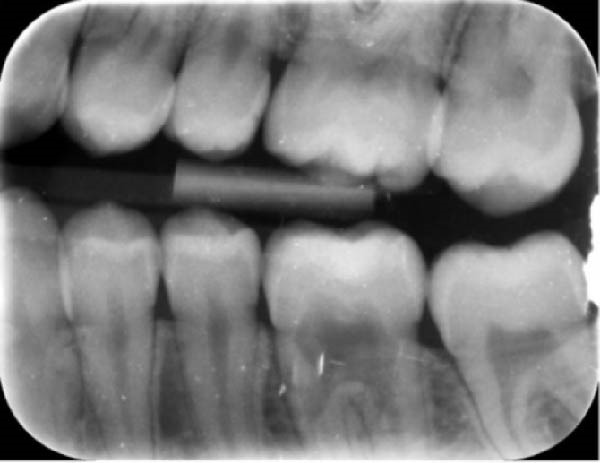
(a)

**Figure:**
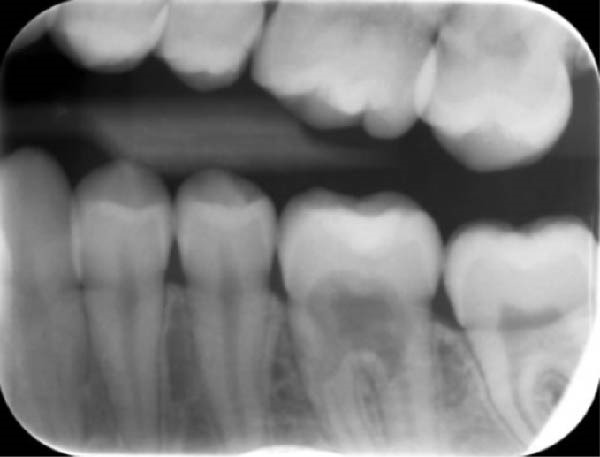
(b)

**Figure:**
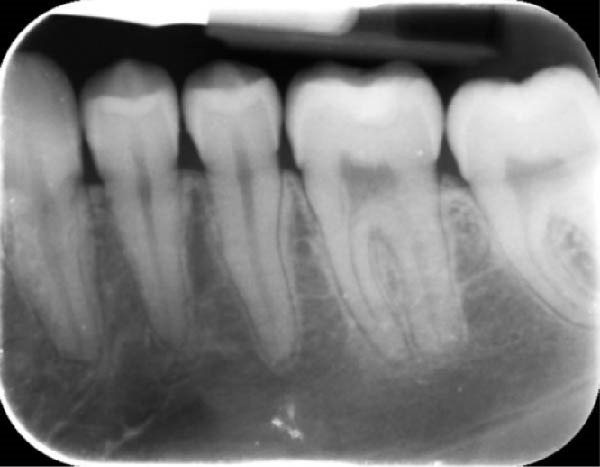
(c)

### 2.2. Patient History

At consultation, the patient reported no symptoms and denied any history of discomfort or previous issues with Tooth 37. She completed fixed orthodontic treatment for the management of a Class II Division 1 malocclusion (including a 7 mm overjet) over 10 years prior, which involved 2 years of fixed orthodontic appliances, with Class II inter‐maxillary traction (Figure 2). A few years prior to this, she had had Tooth 36 extracted, for reasons she could not recall. Following completion of the orthodontic treatment, the patient wore removable retainers nightly for 2 years, after which she stopped. She was unaware of any parafunctional habits and denied any history of dental trauma or tooth bleaching. She had always had pet cats in her family.

Figure 2Clinical photographs and radiograph from the patient’s orthodontic records (published with the patient’s consent). (a) Pre‐orthodontic panoramic radiograph from 22/01/2014 demonstrating the absence of Tooth 36 at baseline; Tooth 37 was fully erupted with no evidence of ECR, and Tooth 38 remained unerupted and under development. (b) Right lateral intraoral view of fixed orthodontic appliances (26/02/2015). (c) Frontal intraoral view of fixed orthodontic appliances (26/02/2015). (d) Left lateral intraoral view of fixed orthodontic appliances (26/02/2015).

**Figure:**
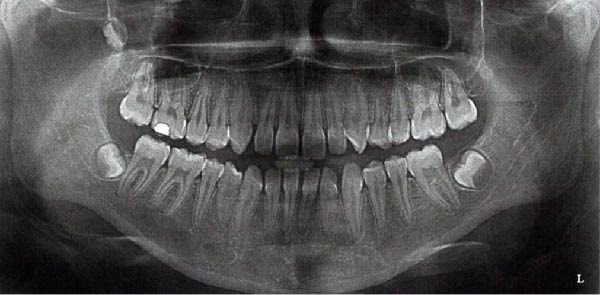
(a)

**Figure:**
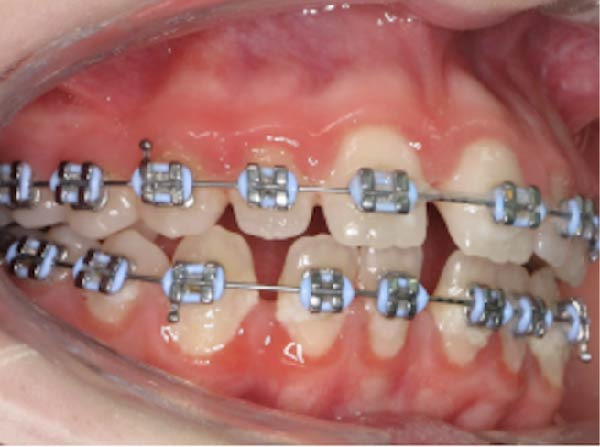
(b)

**Figure:**
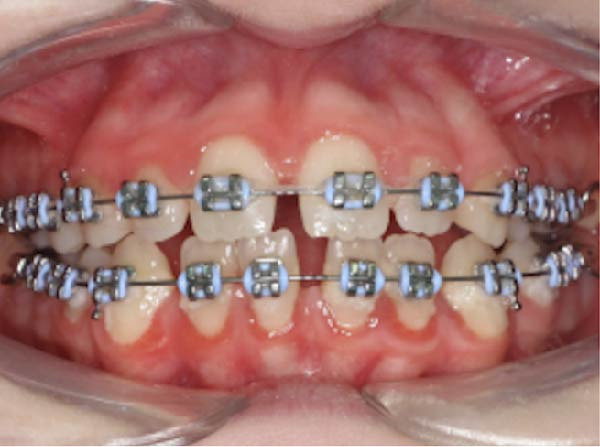
(c)

**Figure:**
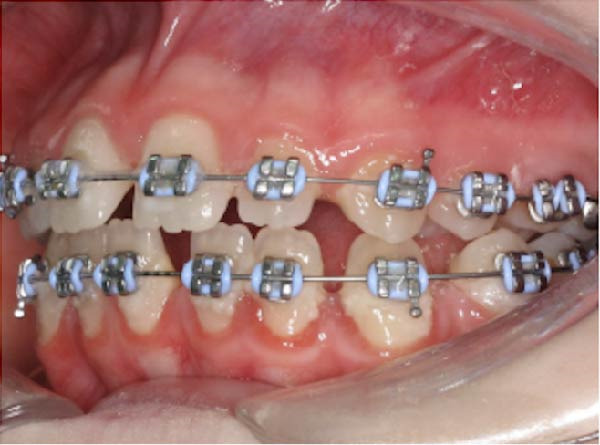
(d)

### 2.3. Medical and Social History

The patient was medically fit and well, with no reported systemic conditions, medications or allergies. The patient was a non‐smoker, and her alcohol consumption was below the recommended limits.

### 2.4. Clinical Examination

Extraoral examination revealed bilateral temporomandibular joint (TMJ) clicking, masseteric hypertrophy and antegonial notching.

Intraoral examination showed fair oral hygiene with localised bleeding on probing but no periodontal pocketing (BPE: 010/121). Tooth 37 had an occlusal composite restoration that was clinically sound (Figure 3). The tooth was not tender to percussion or palpation. There was no evidence of swelling, sinus tract or isolated periodontal pocketing. Thermal pulp sensibility testing (Endo‐Frost) elicited positive responses from Teeth 35–38, all within normal limits.

Figure 3Two‐dimensional radiographic, CBCT, and clinical assessment at the 8‐year follow‐up (13/05/2025), following the initial incidental detection of an external cervical resorption (ECR) lesion. (a, b) Right and left bitewing radiographs demonstrate alveolar bone levels within normal limits, with no radiographic evidence of caries. Tooth 37 displays a pulp chamber of normal size and morphology. A possible enlargement of the pulp chamber in Tooth 26 was noted; however, this could not be definitively evaluated on two‐dimensional imaging. (c) A periapical radiograph of Tooth 37 confirms the findings of the bitewing radiographs, showing a normal pulp chamber outline and no signs of periapical pathology. (d–h) Medium‐volume and high‐resolution CBCT imaging of Teeth 37 and 26, including adjacent structures, confirms the presence of an ECR lesion on Tooth 37. Regions of increased radiodensity suggest reparative tissue activity; however, two portals of entry remain visible—one mid‐buccally and one disto‐buccally (yellow arrows). An additional ECR lesion is identified on Tooth 26 (red arrows), classified as Patel [14] Class 2Ap. Periapical tissues associated with both teeth appear normal on three‐dimensional evaluation. (i–k) Intraoral clinical photographs taken at the 8‐year follow‐up reveal a well‐maintained, minimally restored dentition. Closer inspection of Tooth 37 reveals the presence of craze lines, potentially indicative of an undiagnosed parafunctional habit such as bruxism or clenching.

**Figure:**
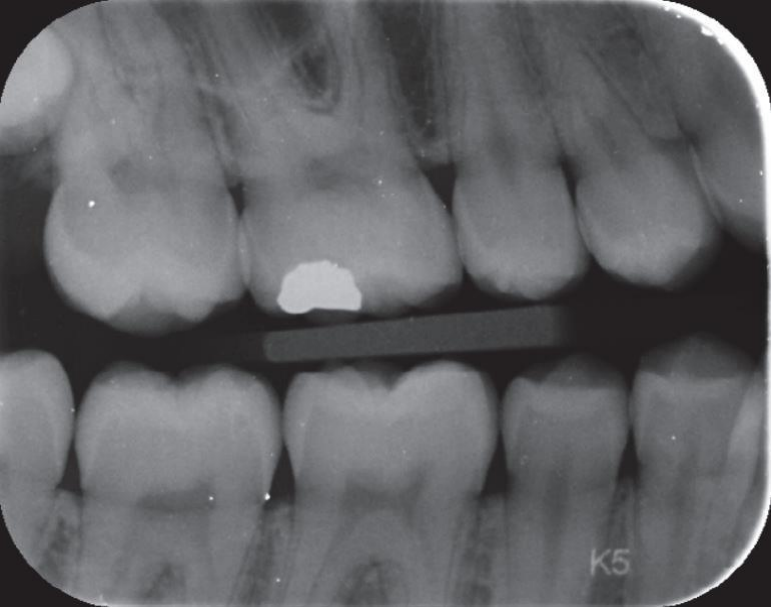
(a)

**Figure:**
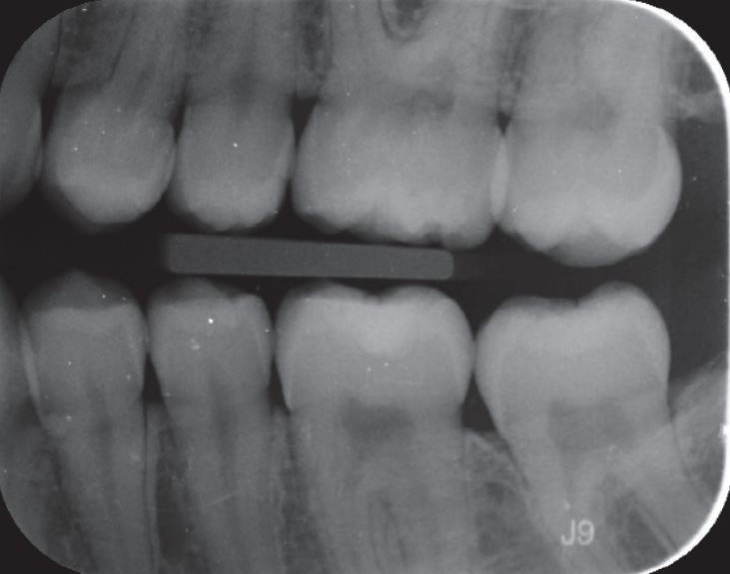
(b)

**Figure:**
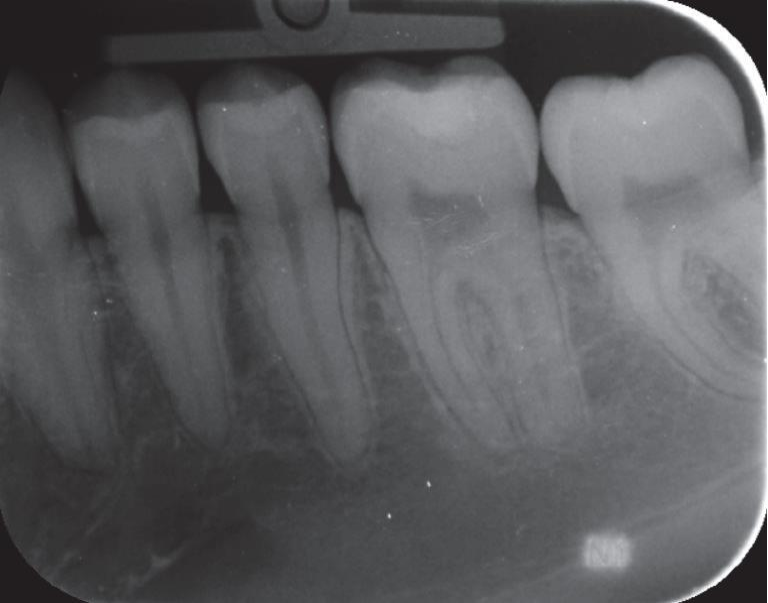
(c)

**Figure:**
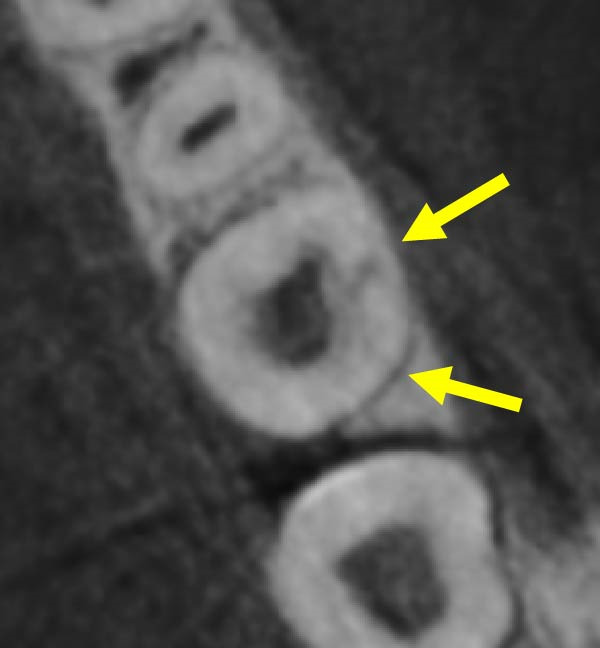
(d)

**Figure:**
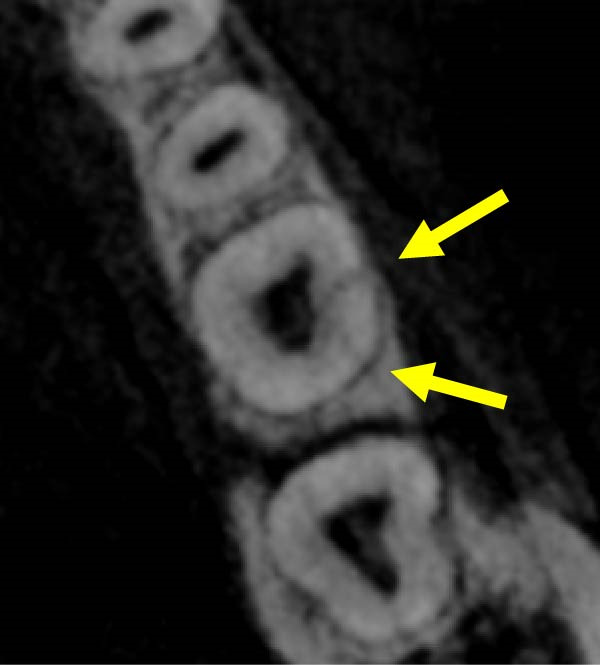
(e)

**Figure:**
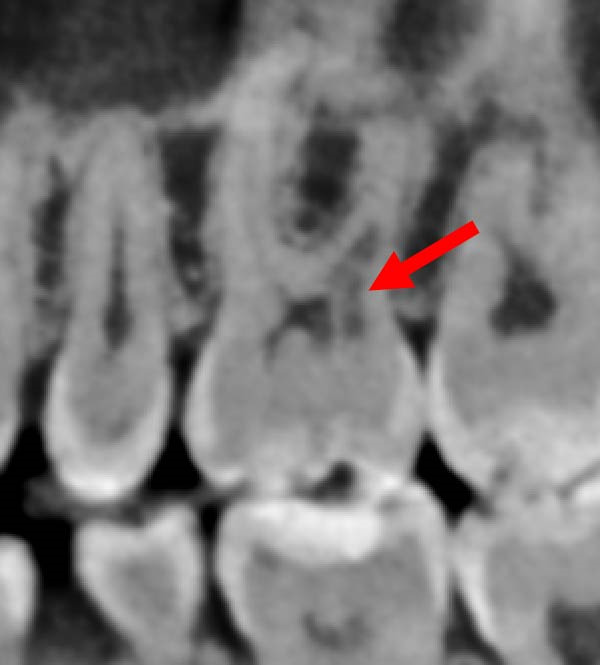
(f)

**Figure:**
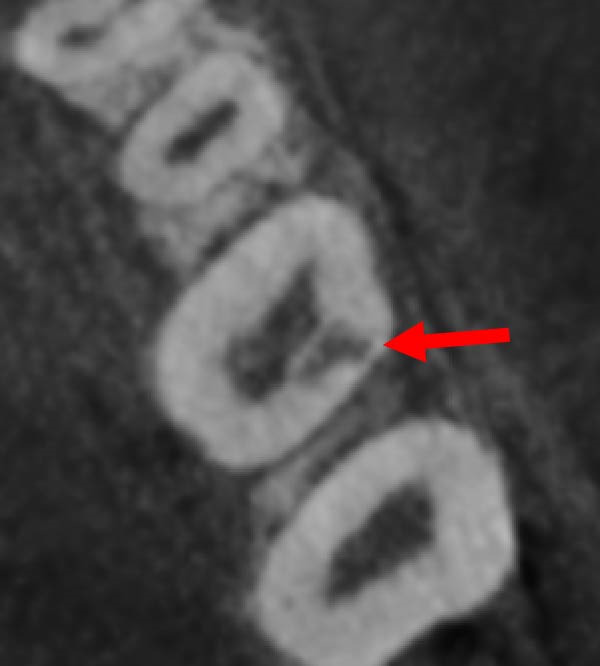
(g)

**Figure:**
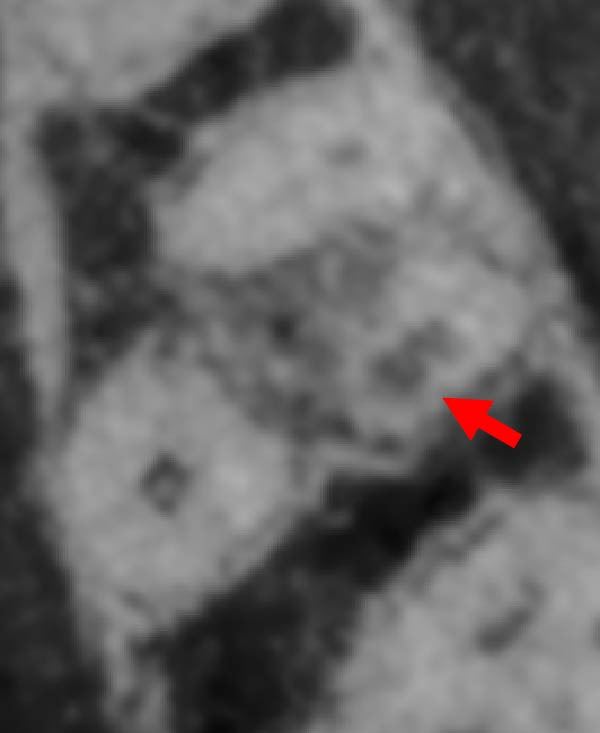
(h)

**Figure:**
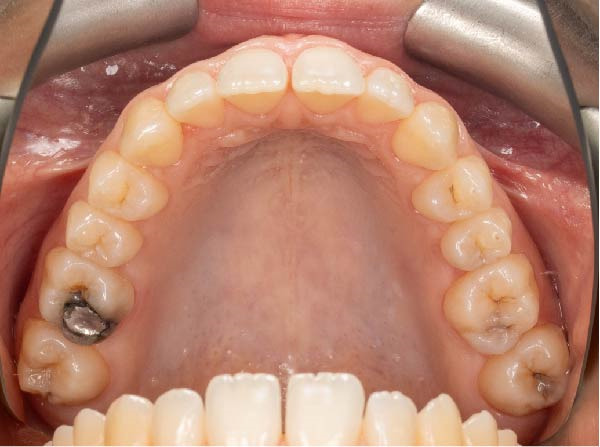
(i)

**Figure:**
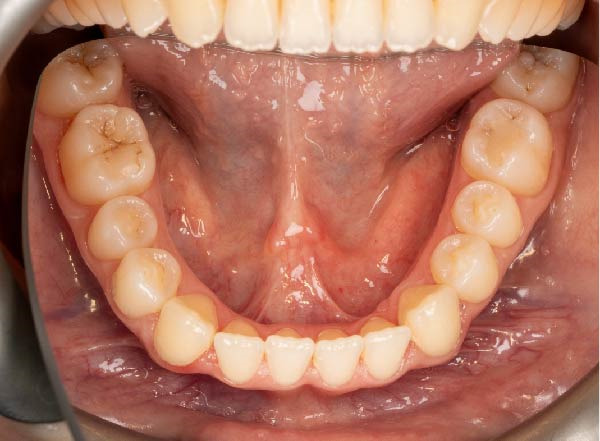
(j)

**Figure:**
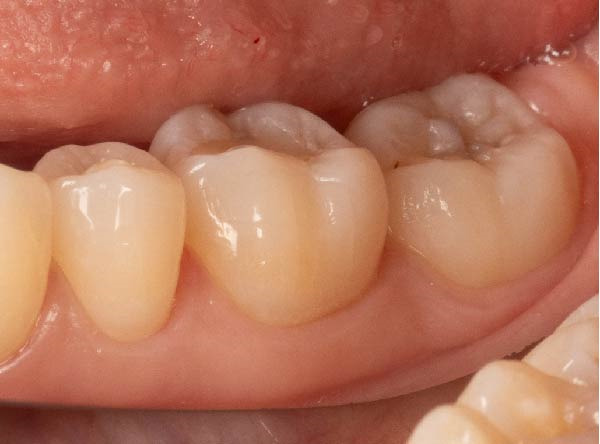
(k)

### 2.5. Two‐ and Three‐Dimensional Radiographic Findings (Figure 3)

Periapical and bitewing radiographs demonstrated normal crestal bone levels. Tooth 37 exhibited a normal pulp chamber with possible coronal canal calcification and no periapical pathology. A CBCT examination was undertaken to provide targeted diagnostic clarification of Tooth 37 and to assess for additional ECR lesions, employing a limited field of view and adhering to current radiation protection guidelines (ALARA/ALADA principles). CBCT imaging demonstrated two radiolucent channels at the cervical region of Tooth 37—one disto‐buccal and one mid‐buccal—extending into the inner third of the dentine. The radiographic appearance was consistent with ECR and was confirmed by a consultant oral and maxillofacial radiologist. Comparison with earlier imaging suggested partial dentine remineralisation. Incidentally, an additional ECR lesion was identified in Tooth 26, with possible pulpal involvement affecting the mesio‐buccal canal and no radiographic evidence of associated periapical pathology.

### 2.6. Diagnoses

1. Localised plaque‐induced gingivitis.
2. Tooth 37 normal pulp and periapical status, with ECR, currently in a reparative phase—Patel et al. [14] classification: 1Bd.
3. Tooth 26 normal pulp and periapical status, with ECR—Patel et al. [14] classification: 2Ap.

### 2.7. Management Plan

Following discussion with the patient, the following plan was agreed upon:

1. Oral hygiene instruction and generalised professional mechanical plaque removal (PMPR).
2. Six‐month review of Tooth 37 by the endodontist.
3. Management options for Tooth 26 included either continued monitoring without active intervention or non‐surgical root canal therapy combined with internal repair of the resorptive defect. In light of the favourable outcome observed in Tooth 37 after long‐term monitoring, the patient elected to manage Tooth 26 similarly: with monitoring.

## 3. Discussion

Prior to proposing a physiological explanation for the present case, confirmation of the ECR diagnosis was essential. The principal differential diagnosis considered was pre‐eruptive intra‐coronal resorption (PEIR), a rare developmental anomaly characterised by a radiolucent defect within the coronal dentine of an unerupted tooth, typically detected incidentally on radiographs [15]. PEIR was excluded for several reasons: CBCT demonstrated a distinct resorptive channel consistent with an ECR portal of entry; the lesion extended beyond the coronal region into radicular dentine and did not mimic caries, unlike PEIR [16]; critically, the pre‐orthodontic panoramic radiograph confirmed a normal Tooth 37, thereby confirming that the resorption had developed post‐eruption. Furthermore, PEIR is not reported to exhibit radiographic evidence of repair, whereas this lesion demonstrated features suggestive of partial remineralisation. Diagnostic interpretation by an oral and maxillofacial radiologist corroborated these findings. Collectively, these considerations confirm the diagnosis of ECR in Tooth 37 beyond a reasonable doubt.

Root resorption is mediated by multinucleated clastic cells, commonly referred to as odontoclasts, which originate from the monocyte–macrophage lineage. These cells possess the capacity to resorb mineralised dental tissues, including dentine and cementum. Although there remains debate about whether odontoclasts are functionally distinct from osteoclasts, they are generally described as smaller, with fewer nuclei and less extensive sealing zones than their osseous counterparts [17–20].

Under physiological conditions, dental hard tissues are protected from resorptive activity by non‐mineralised surface layers, namely, predentine on the pulpal aspect and precementum on the external root surface. These layers act as protective barriers, preventing clastic cell attachment. This protective effect has been attributed to inhibitory factors present within the organic matrices that limit cellular adhesion [21–23]. Furthermore, successful attachment of clastic cells requires interaction with extracellular matrix proteins containing the arginine–glycine–aspartic acid (RGD) sequence. These adhesion molecules are abundant in mineralised tissues but largely absent from predentine and precementum, further reducing the likelihood of resorption under normal conditions [24]. In addition, the presence of odontoblasts internally and cementoblasts and fibroblasts externally effectively occupies the root surface, restricting access for clastic cells.

Disruption of these protective structures permits clastic cell attachment to exposed mineralised surfaces. Once attachment occurs, integrin receptors on the clastic cell membrane, particularly αvβ3 integrins, bind to RGD–containing proteins, forming a specialised sealing zone. This is accompanied by cytoskeletal reorganisation and the development of a ruffled border, beneath which resorptive activity is concentrated [24]. Within this microenvironment, hydrogen ions are actively transported to acidify the local area, facilitating dissolution of hydroxyapatite crystals. This process lowers the pH to approximately 4.5, enabling demineralisation of the tissue’s inorganic component [25, 26]. The exposed organic matrix is subsequently degraded by proteolytic enzymes, including tartrate‐resistant acid phosphatase, cathepsin K and matrix metalloproteinases [27, 28], resulting in the formation of characteristic resorption lacunae.

Sustained resorptive activity is dependent on the continued presence of a stimulus. In the absence of such stimulation, clastic activity diminishes and reparative processes may ensue, characterised by deposition of cementum‐ or bone‐like tissue. This transient resorption phase contrasts with progressive resorption, in which ongoing mechanical or inflammatory stimuli perpetuate clastic activity, leading to continued tissue loss.

In the present case, exposure of cervical dentine may have arisen either from a pre‐existing anatomical variation at the cemento‐enamel junction or as a consequence of iatrogenic damage during orthodontic treatment. Anatomical studies have demonstrated variability in the cemento‐enamel junction, with enamel overlapping cementum in approximately 60% of cases, a butt joint present in around 30%, and a gap exposing dentine in approximately 10% of individuals [29]. Even in the absence of a naturally exposed dentine surface, orthodontic procedures, particularly band placement, may result in localised damage and subsequent exposure of susceptible tissue [1].

Orthodontic forces may also contribute to the initiation of resorption through vascular and cellular mechanisms. Excessive or prolonged forces can compromise the blood supply within the periodontal ligament, leading to localised hypoxia and tissue necrosis [4, 13]. Emerging evidence suggests that hypoxic conditions may promote differentiation of precursor cells into clastic phenotypes, thereby facilitating resorptive activity [30–33]. These mechanisms provide a plausible explanation for the initiation of ECR in susceptible individuals, including the subject of this case report.

Following initiation, ECR lesions may exhibit both resorptive and reparative phases. The resorptive phase involves progressive infiltration of clastic cells through dentine, often advancing towards the pulp. The pericanalar resorption‐resistant sheet (PRRS), a protective layer composed of dentine and predentine, may initially limit pulpal involvement, although this barrier can be compromised in more advanced lesions [8]. During the reparative phase, deposition of bone‐like or cementum‐like tissue occurs within the defect and remodelling may take place over time [34]. Patel and colleagues [35] reported that ECR lesions are observed in the resorptive and reparative phases in approximately 70.2% and 29.8% of cases, respectively, and may even occur simultaneously within different regions of the same lesion. When ECR enters the reparative phase, the extent of repair detectable on radiographic assessment is generally limited; cases exhibiting radiographic signs of remineralisation of the magnitude observed in the present report are exceptionally rare.

**Figure 4:**
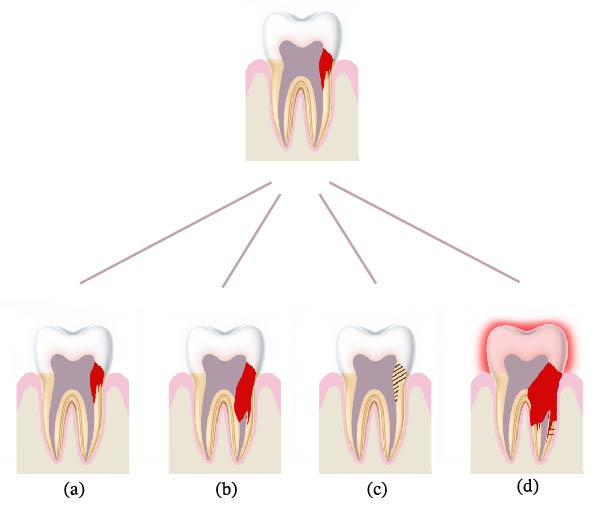
Potential outcomes following the clinical decision to monitor an extensive external cervical resorption (ECR) lesion. (a) No change: the tooth appears clinically and radiographically stable at follow‐up. (b) Progression: increased radiolucency is observed, indicating further resorption, but the tooth remains asymptomatic. (c) Repair: increased radiopacity suggests reparative changes. (d) Deterioration: radiographic evidence of progression (increased radiolucency) is accompanied by clinical signs or symptoms, such as pain or aesthetic concerns due to discolouration.

In this report, a conservative monitoring strategy was employed for a Heithersay Class 3 ECR lesion. Mavridou et al. [36] proposed a clinical decision‐making framework that integrates not only lesion extent and location, as described in prior classifications [13, 14], but also clinical indicators such as probing accessibility, symptomatology and the presence of bone‐like tissue within the lesion. They suggested monitoring may be appropriate when probing is not feasible, symptoms and aesthetic concerns are absent and/or bone‐like ingrowth is present—criteria applicable to the current case. Four potential outcomes of monitoring an ECR lesion were described: (1) no clinical or radiographic progression; (2) no clinical signs/symptoms but increased radiolucency, indicating progressive resorption; (3) no clinical signs/symptoms but increased radiopacity consistent with reparative deposition—as observed in our patient; (4) progression with clinical (e.g., pain or discolouration) and/or radiographic signs necessitating extraction (Figure 4). Accordingly, long‐term monitoring remains the planned course for this patient.

During the monitoring phase, a comprehensive assessment of all teeth is essential, as additional ECR lesions may arise, as exemplified by the involvement of Tooth 26 in the present case. This report further highlights the superior diagnostic sensitivity of CBCT compared with conventional two‐dimensional radiography for detecting ECR.

This report has several limitations. Histopathological confirmation of the nature of the hard‐tissue deposition was not available, and interpretation was, therefore, based exclusively on conventional radiographic and CBCT findings. Accordingly, the term ‘repair’ is used in a strictly radiographic context, and it cannot be excluded that the observed hard tissue represents bone‐ or cementum‐like replacement rather than complete restitution of the original dentine architecture.

## 4. Conclusion

Although limited to a single case, this report highlights the potential for reparative outcomes in asymptomatic ECR lesions initially deemed to have a poor or hopeless prognosis. In select cases where intervention is impractical, and the resorptive stimulus may have ceased or been mitigated, long‐term monitoring could serve as a viable alternative to immediate extraction, particularly when radiographic and clinical stability is maintained.

## Author Contributions

**Farnam Pourreza-Jorshari**: conceptualisation, investigation, data curation, formal analysis, visualisation, writing – original draft, writing – review and editing. **Georgios Patouris**: validation, writing – review and editing. **Faraz Pourreza-Jorshari**: visualisation, software, writing – review and editing. **Lee Feinberg**: validation, formal analysis, writing – review and editing. **Shalini Kanagasingam**: supervision, validation, writing – review and editing.

## Funding

This study did not receive any specific funding.

## Disclosure

All authors have read and approved the final version of the manuscript. Farnam Pourreza‐Jorshari had full access to all of the data in this study and takes complete responsibility for the integrity of the data and the accuracy of the data analysis.

## Ethics Statement

This study describes a single anonymised patient case and therefore did not require formal ethical approval in accordance with institutional and national guidelines. Written informed consent was obtained from the patient for publication of this case report and any accompanying images. All procedures were conducted in accordance with the principles outlined in the Declaration of Helsinki.

## Conflicts of Interest

The authors declare no conflicts of interest.

## Data Availability

The authors confirm that the data supporting the findings of this study are available within the article.
